# Population frailty trends by education and income levels over a period of 30 years: findings from Swedish registry data

**DOI:** 10.1136/jech-2023-221060

**Published:** 2023-10-03

**Authors:** Alexandra Wennberg, Yining Tao, Stina Ek, Karin Modig

**Affiliations:** Unit of Epidemiology, Institute of Environmental Medicine, Karolinska Institutet, Stockholm, Sweden

**Keywords:** mortality, aging, epidemiology

## Abstract

**Background:**

Frailty is an age-related health condition affecting an estimated 18% of older adults in Europe and past evidence has shown a relationship between socioeconomic factors and frailty. We examined population frailty trends and the association between frailty and 5-year mortality by education tertiles and income quartiles at ages 75, 85 and 95 in Swedish registry data.

**Methods:**

All Swedish residents born in 1895–1945 and in the Total Population Register from 1990 to 2020 were included. Frailty was assessed with the Hospital Frailty Risk Score (HFRS), which sums 109 weighted International Classification of Diseases (ICD codes), collected from the National Patient Register.

**Results:**

Regardless of education and income, frailty increased over time, though the association between frailty and 5-year mortality remained stable. Particularly in earlier birth cohorts, although the highest education and income levels had the highest mean HFRS scores, the lowest education and income levels accounted for greater proportions among the frail. These trends varied slightly by sex and age. Men and women had similar levels of frailty, but frailty was more strongly associated with mortality among men.

**Conclusion:**

Over time, education and income levels were more equally represented among the frail population in more recent years. More equitable distribution over time may suggest improvement in health disparities, though more work is needed. The overall increase in frailty and unchanged association with mortality indicates that additional research is needed to better understand how to best support the growing ageing population. This would then support the long-term viability of the healthcare system.

**WHAT IS ALREADY KNOWN ON THIS TOPIC**

- Frailty is becoming more common as the population ages, and studies have shown an association between some markers of socioeconomic status (SES) and frailty. However, how these trends and associations may have changed over time, as societies have shifted culturally, economically and relationally, is not known.

**WHAT THIS STUDY ADDS**

- How frailty trends have changed over 30 years in relation to SES, as measured by education and income level. Overall, we observe a more equitable distribution of frailty in the population, though sex differences persist and the association with mortality has remained stable.

**HOW THIS STUDY MIGHT AFFECT RESEARCH, PRACTICE OR POLICY**

- Considering the increase in frailty across all SES strata and unchanged association between frailty and mortality, research and policy work are needed to understand how to support older adults and the long-term viability of the healthcare system.

## Introduction

Frailty is an age-related condition affecting an estimated 18% of older adults in Europe, though prevalence estimates vary widely (2%–75%) by country, setting and frailty measure used.^1^ It is a clinical syndrome characterised by functional decline that increases an individual’s vulnerability to loss of independence, disability and death.^2 3^ Frailty has a complicated, heterogeneous aetiology and is sometimes difficult to differentiate from multimorbidity. With the population ageing, more individuals are at risk of becoming frail. Indeed, we recently found that frailty has been increasing over time in the Swedish population, though the association between frailty and mortality has not changed.^4^

Although our previous work found an increase in frailty over time, an understanding of what the frail population looks like in terms of socioeconomic factors is critical for providing us with information about how healthcare systems need to evolve to meet the needs of an ageing society.^5^ Moreover, in our previous study, we observed that despite increasing levels of frailty, the relationship between frailty and mortality did not substantially change over time, nor did it differ by sex. Whether this is also true for difference socioeconomic status (SES) groups is not known. Socioeconomic trends have also changed over time, as societies have shifted culturally, intellectually, economically and relationally, and this impacts health.^6^ SES impacts frailty prevalence, though different markers of SES (eg, education, income) impact frailty risk differently.^7^ SES markers may follow different patterns of impacting risk throughout the life course (eg, convergence, continuity or maintenance or divergence). The convergence theory posits that SES-associated effects peak in early or middle age and that disparities are reduced over time with no significant differences between SES groups after age 85, because of the high mortality among low SES groups, universal age-related frailty and benefits of welfare policies.^8 9^ Continuity posits that SES-associated effects are established in early or middle age, and the differences between strata are maintained from that point forward.^9 10^ Finally, divergence posits that people in lower SES groups face various challenges throughout their lives (eg, food insecurity, housing insecurity, etc) that cause health inequality to accumulate over the life course.^9 11^ However, how time trends may have impacted the relationship between SES and frailty remains relatively understudied. With socioeconomic changes in society, it may be that there is a trend towards equalisation over time.

To understand frailty trends by SES, this descriptive study examined frailty by education and income in the entire population of Sweden from 1990 to 2020 at ages 75 (birth cohorts 1915–1945), 85 (birth cohorts 1905–1935) and 95 (birth cohorts 1895–1925). Three ages were chosen to give an overview of frailty trends over time at different stages of older adulthood. We additionally examined the association between frailty and 5-year mortality. Finally, due to the sex-frailty paradox,^12^ we a priori chose to additionally conduct sex-stratified analyses, to determine how sex may modify frailty trends by SES.

## Methods

### Data and participants

We used Swedish register data, including the Total Population Register (TPR), the National Patient Register (NPR), the Longitudinal Integrated Database for Health Insurance and Labour Market Studies (LISA) and the Cause of Death Register. The Swedish personal identity number provides linkage across databases at the individual level and provides follow-up of all residents with essentially zero attrition. We included Swedish residents born between 1895 and 1945 and in the TPR from 1990 through 2020. Sociodemographic and medical data were collected for each individual for the year leading up to their date of birth at ages 75, 85 and 95. This provided a cross-sectional assessment of frailty at ages 75, 85 and 95 from 1990 to 2020.

This research was register based and did not involve direct contact with the study participants, thus informed consent was not required.^13^

### Frailty score and mortality

We assessed frailty trends across birth cohorts and examined the association between frailty and mortality. Frailty was assessed with the Hospital Frailty Risk Score (HFRS), which is based on the cumulative deficit model of frailty and sums the primary and secondary diagnostic codes from an individual’s medical records. The cumulative deficit model of frailty defines frail individuals through the accumulation of health conditions and/or functional limitations, such as in the gold-standard Rockwood Frailty Index,^14^ though this inherently has overlap with measures of multimorbidity. The HFRS score is based on the weighted (0.1–7.1) sum of 109 ICD-10 codes.^15^ We obtained ICD codes from the NPR including inpatient, outpatient and specialist (from 2011) care.^16 17^ ICD codes were translated into Swedish diagnostic codes (online supplemental table 1). The HFRS is a continuous score, but it can be categorised, using cut-points at 5 and 15 to define frail and highly frail individuals.^15^ Individuals who did not have codes in the NPR for the year leading up to their date of birth at ages 75, 85 and 95 were assigned an HFRS score of 0 (online supplemental table 2). These individuals were not hospitalised or did not seek specialised outpatient care, though they may still have sought primary care. We conducted analysis including the entire population and analysis excluding those who were not hospitalised and had not received specialised outpatient care. All-cause mortality and date of death were derived from the Cause of Death Register.

10.1136/jech-2023-221060.supp1 Supplementary data

### Sociodemographic factors

Sex was collected from the TPR, while education and income were collected from LISA.^18^ Education was grouped into tertiles defined as ≤9 years, 10–12 years and>12 years based on the number of years of formal schooling. In birth cohorts from 1895 to 1910, all education data were missing, so these birth cohorts were excluded from the education analyses but included in income analyses. Income was divided into quartiles (25th, 50th, 75th, 100th) based on the individual disposable income ((sum of disposable income for all family members, inclusive of pension×the individual’s consumption weight)/family’s total consumption weight) each year, thus, the quartiles were dynamic and accounted for both household size and inflation.^19^

### Statistical analysis

We first examined frailty trends overall, by sex and across education tertiles and income quartiles at ages 75, 85 and 95 ages by plotting a linear graph and stacked area chart. The linear graphs plotted the mean HFRS score both among all people in the population registers and excluding individuals who had an HFRS score of 0, indicating that they did not seek inpatient or outpatient healthcare for that specific year. The percentage stacked area charts represented the relative distribution trends of frailty (proportion of frail population) by education and income. We used HFRS cut-points of 5 and 15, representing the frail and highly frail populations, respectively. Cox proportional hazard models were used to examine the association between frailty and 5-year mortality. We estimated hazard ratios for men and women, education tertiles and income quartiles across birth cohorts at all three ages. The timescale was defined as the time between the year frailty was assessed (at age 75, 85 or 95 years) and the following of 5 years. Given this timescale and to exclude COVID-19-related mortality effects, participants who reached 75, 85 and 95 years after 2014 were excluded from this analysis. All statistical analyses were completed with Stata V.16 (StataCorp LLC, College Station, Texas, USA).

## Results

Overall, regardless of education or income, we observed an increase in mean HFRS among both the total population and the population that sought specialist care or hospital care across birth cohorts at ages 75, 85 and 95 and that, unsurprisingly, women accounted for a greater proportion of the frail population at older ages. Among the total population (including individuals with an HFRS score of 0), the average frailty score from 1990 to 2020 ranged from 0.25 to 0.77 at age 75, from 0.66 to 1.65 at 85 and from 0.83 to 2.49 at 95. Excluding individuals not present in the NPR, with an HFRS score of 0, the HFRS ranged from 2.66 to 3.56, from 3.16 to 4.97 and 3.03 to 5.93 at ages 75, 85 and 95, respectively (online supplemental figure 1). In graphing the distribution of the frail population, we found that there were fewer people who were very frail in earlier birth cohorts compared with later birth cohorts. The proportion of frail people ranged from 0% to 10% at age 75, from 0% to 15% at age 85 and from 0% to 14% at age 95 (online supplemental figure 2). The lack of very frail people in earlier birth cohorts may be indicative of changes in diagnosis settings or improved disease survival.

### By education tertiles

**Figure 1:**
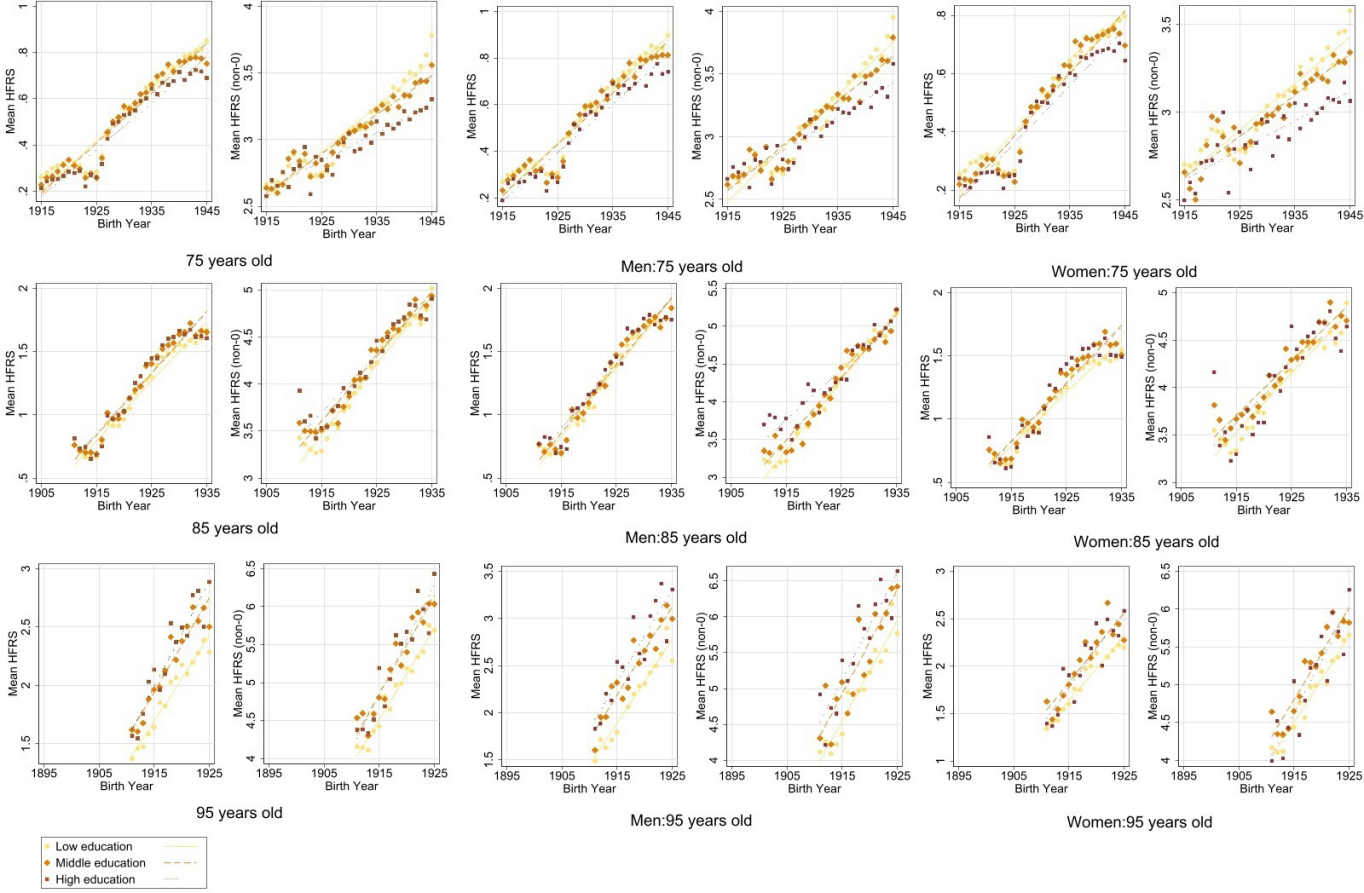
Mean Hospital Frailty Risk Score (HFRS) (left: include 0; right: exclude 0) with fitted line by education level among total population, men and women at ages 75, 85 and 95 in birth cohorts 1895–1945, corresponding to calendar years 1990–2020.

**Figure 2:**
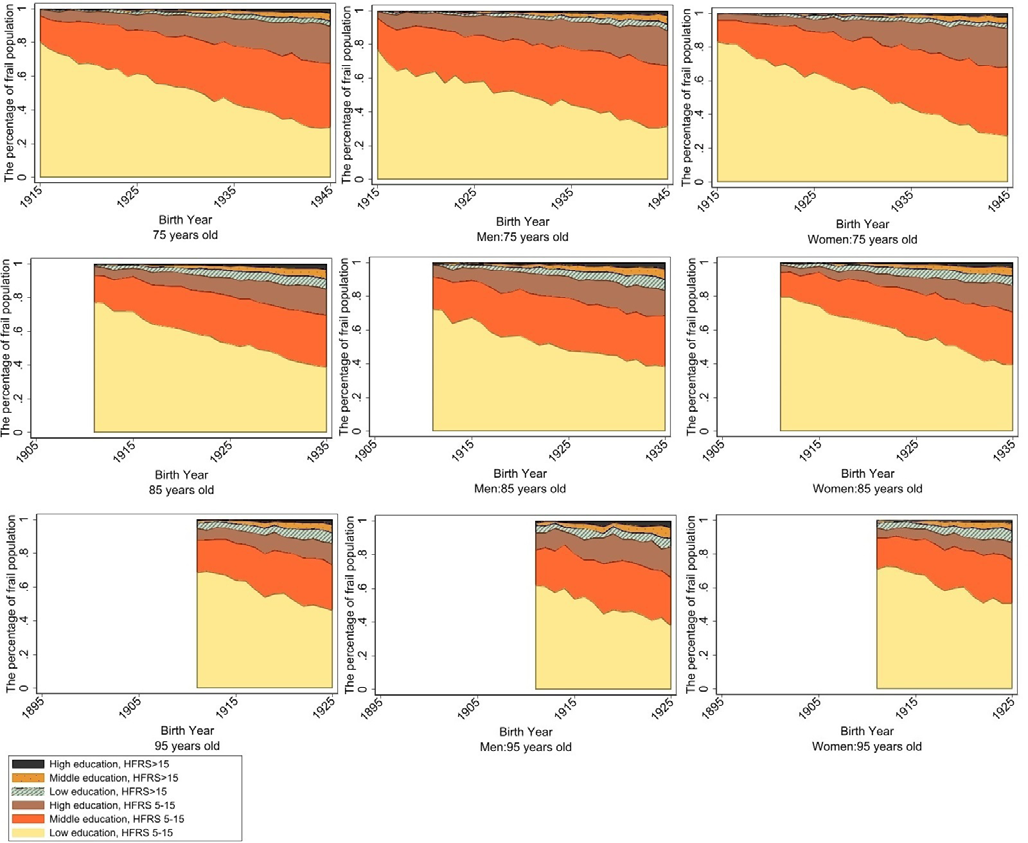
Proportion of low, middle and high educational attainment represented among the frail and highly frail for the total population, men, and women at ages 75, 85, and 95 in birth cohorts 1895–1945, corresponding to calendar years 1990–2020. HFRS, Hospital Frailty Risk Score.

When stratifying by education level, the lowest tertile had the highest mean HFRS at age 75 (0.85). However, at older ages, we observed a different pattern, whereby the middle tertile had the highest mean HFRS at age 85 (1.73) and the highest tertile had the highest mean score at age 95 (2.89) (figure 1). This suggested an age-dependent education trend for HFRS, and the same pattern was observed in the NPR population and in both women and men in sex-stratified analyses. The lowest tertile accounted for the greatest proportion of the frail population at all three ages (average 53%, 57% and 58%, respectively) (figure 2). However, when examining trends across birth cohorts, we observed that in more recent birth cohorts, the lowest tertile was increasingly less represented among the frail population, with more equitable distribution among the education tertiles. For example, at age 75, the proportion decreased from 80% to 29%, at age 85 from 77% to 39% and at age 95 from 69% to 46%. This decreasing trend across birth cohorts may be impacted by the decreasing proportion of lower educated people in the total population (online supplemental table 3).

### By income quartiles

**Figure 3:**
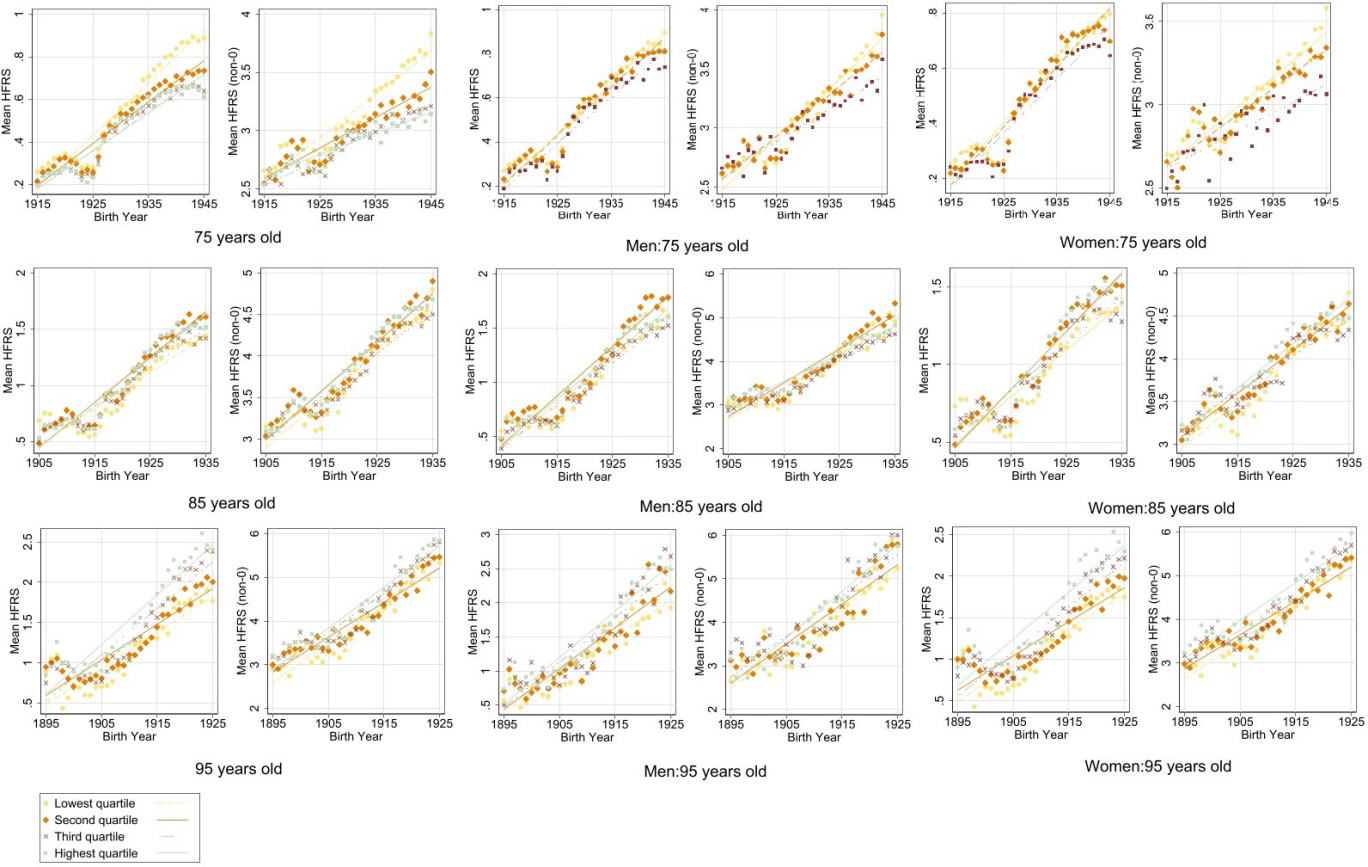
Mean Hospital Frailty Risk Score (HFRS) (left: include 0; right: exclude 0) with fitted line by income quartiles among the total population, men and women at ages 75, 85 and 95 in birth cohorts 1895–1945, corresponding to calendar years 1990–2020.

**Figure 4:**
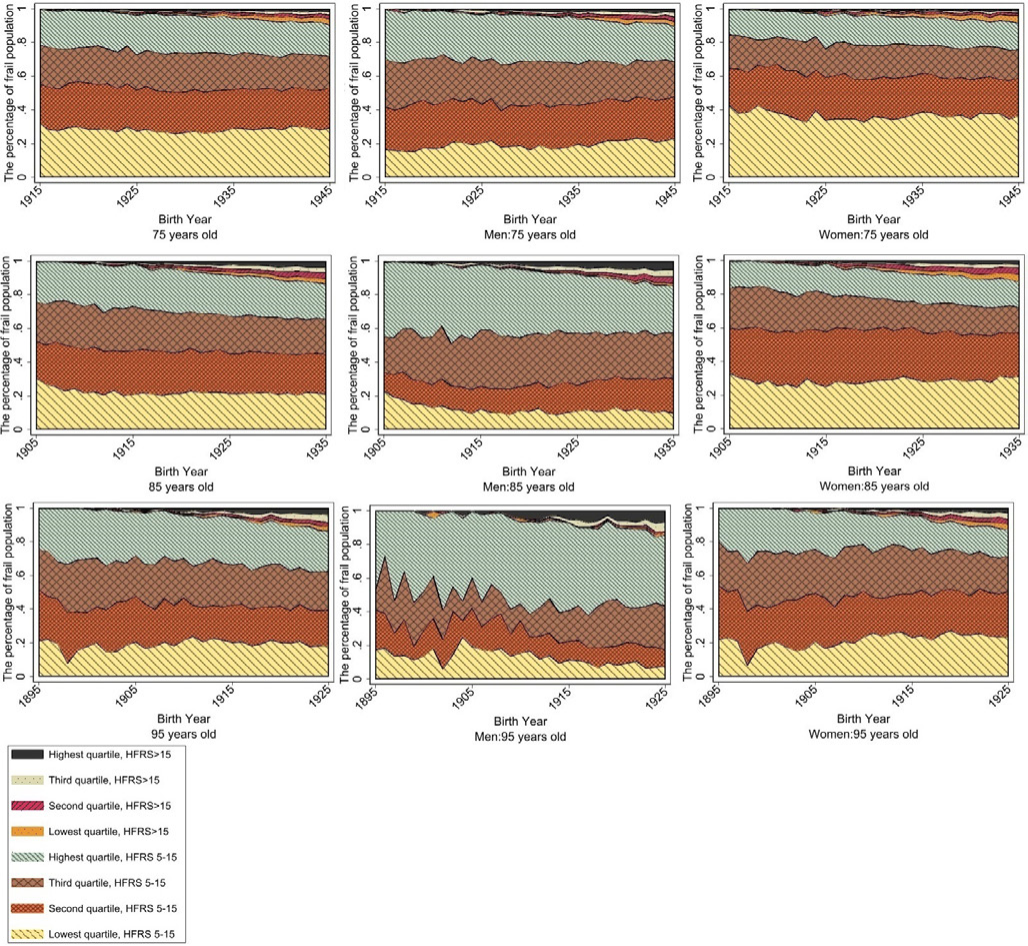
Proportion of 25th, 50th, 75th and highest income quartiles represented among the frail and highly frail for the total population, men and women at ages 75, 85 and 95 in birth cohorts 1895–1945, corresponding to calendar years 1990–2020. HFRS, Hospital Frailty Risk Score.

Higher mean HFRS was observed among the lowest income quartile at age 75 (mean=0.89 and mean=3.8 among the total and NPR populations, respectively) (figure 3). However, at ages 85 (mean=1.56 and mean=4.68 among the total and NPR populations) and 95 (mean=2.88 and mean=6.43 among the total and NPR populations), the highest income quartile had the highest mean HFRS. Figure 4 illustrates the distribution by income quartiles, in which the proportion of the frail population was evenly distributed between income levels at all three ages (figure 4). In analyses stratified by sex, men in the highest income quartile accounted for the greatest proportion of the frail population at ages 85 and 95; though this trend showed a slight decline over time.

### Five-year mortality

**Figure 5:**
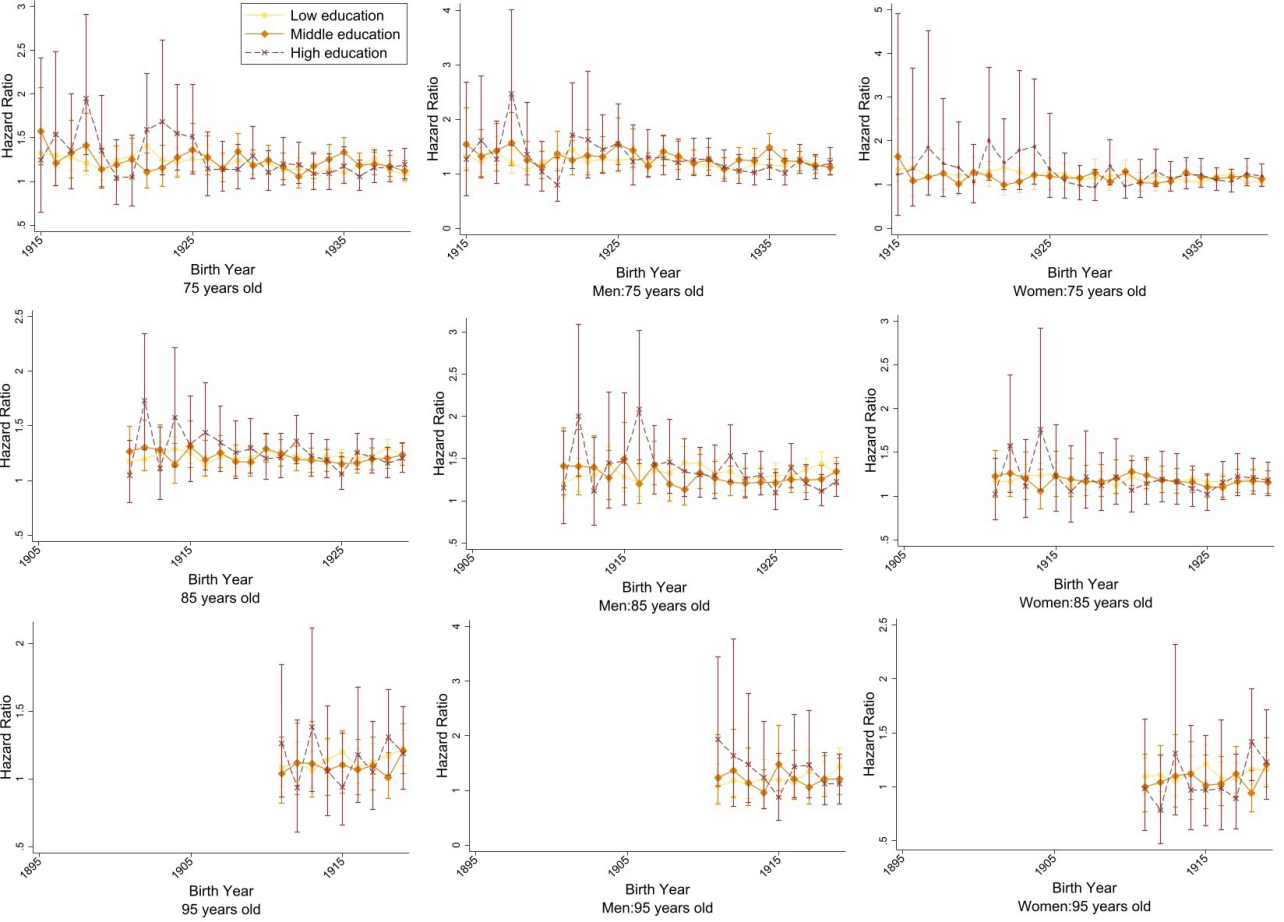
Association between frailty and 5-year mortality by education tertiles for the total population, men and women at ages 75, 85 and 95 in birth cohorts 1895–1945, corresponding to calendar years 1990–2020.

In Cox proportional hazard models, frailty-mortality associations were relatively stable across birth cohorts, regardless of sex, education or income. Differences by education and income were somewhat greater in earlier birth cohorts (though still with overlapping CIs), but trends converged over time. HR estimates remained stable in both sexes at all three ages, though the absolute mortality trend among men was higher than among women (online supplemental figure 3). Among the low and middle education tertiles, the association between frailty and mortality was similar and followed a similar pattern across birth cohorts at ages 75, 85 and 95 (figure 5). Among the highest education tertile, the trends fluctuated moderately. In all education tertiles, the association between frailty and mortality remained fairly consistent over time. In analyses additionally stratified by sex, men showed a stronger association between frailty and mortality in the highest education tertile, and this was particularly true in earlier birth cohorts. There were no substantial differences in HR trends over time comparing different income quartiles (online supplemental figure 4).

## Discussion

Here, we have shown how frailty increased over time at all studied ages, for men and women, and that frailty has become distributed more equitably across SES. The relative association between frailty and mortality has remained stable over time and is relatively similar across SES groups. However, men, as compared with women, have higher relative mortality risk given the same levels of frailty. This is consistent with recent findings of multimorbidity and mortality from large cohort studies showing that although men and women had similar multimorbidity burden, it was more strongly associated with mortality among men.^20^ Similarly, women report greater physical function impairment, but it is more strongly associated with mortality in men.^21^ This may partially be explained by findings showing that women have healthier lifestyles and are more likely to report health issues with more detail and accuracy, perhaps leading to more effective treatment of these conditions.^22 23^ Studies have also shown that psychosocial factors are strong determinants of health among women, while physical health conditions are stronger determinants of health among men.^24^

We found that the lowest education tertile accounted for the greatest proportion of frailty at all three ages, consistent with previous studies.^9 25^ This is in line with the continuity hypothesis, that an early gap between educational groups could be maintained from earlier to later life.^9 10^ This is supported by a recent meta-analysis examining the association between education and frailty.^7^ However, over time, we observed that the lowest tertile accounted for a smaller proportion of the frail population at all three ages. In 1990, those in the lowest tertile accounted for approximately 80% of the frail population at all three ages but by 2020, the lowest education tertile accounted for approximately 40% of the frail population. There were no differences between education tertiles when examining the association between frailty and 5-year mortality. This is consistent with a recent study that found that education was not associated with the transitions from healthy state to frailty (as well as multimorbidity) to mortality.^26^ Possibly, a more equitable distribution of resources, including healthcare, education and improved modifiable lifestyle factors, has led to improved health for people with lower SES. This has been supported by Swedish health policies in more recent decades to create better health equality.^27^ Notably, the mean frailty score by education level was different at different ages. At age 75, the lowest tertile had the highest mean HFRS; at ages 85 and 95, the higher tertiles had the highest mean HFRS. This may indicate a survival selection between the educational groups. People with higher educational attainment are more likely to live to older ages, which may mean that those with lower educational attainment who live longer are healthier than the lower educated group as a whole at a younger age.^9^ However, missing education data in earlier birth cohorts may have affected this pattern, particularly at ages 85 and 95, and the results should be interpreted with caution.

The education findings are slightly contrasted to the income findings, where the quartiles have been more equally represented among the frail population over time. Notably, there has been a decrease in the proportion of the highest income quartile over time, which accounted for a greater proportion of the frail population earlier. This is more in line with the convergence hypothesis than the continuity hypothesis and has been supported by evidence from a study investigating income and frailty in ten European countries^9^ and in a recent meta-analysis that also found that the majority of studies investigating SES and frailty show that income is a leveller across the life course.^7^ This may be even more relevant in a country such as Sweden, where welfare and governmental supports are strong. Comparing education and income, studies have shown that education is more strongly associated with frailty than income (and occupation and wealth, too).^9^

Additionally, we observed different patterns between men and women. Men at higher income levels accounted for the largest percentage of the frail population at ages 85 and 95, whereas the income quartiles in the frail population were more equally distributed among women, consistent with previous work.^5^ This may be in part a reflection of generational differences, where women in earlier cohorts were more reliant on their husband’s income, thus creating sex differences in the results.^18^ However, this may also reflect differences in life expectancy and health in older men and women. Men and people with lower SES have shorter life expectancies,^12^ so it may be that men in lower income groups are less likely to survive to older ages and the men in lower income groups who do survive to older ages are healthier. Regardless, we did not observe a difference in the association between frailty and 5-year mortality by income level, even considering sex as an effect modifier. This is consistent with findings showing no stronger association between frailty or multimorbidity and mortality among lower SES groups.^26^

The primary strength of this study is the inclusion of all Swedish residents over a 30-year period, which provided us with a large scope to investigate frailty trends. However, the limitations of the study must also be considered when interpreting the results. The HFRS relies on diagnostic codes, and we were not able to account for diagnoses in primary care, this would result in overall lower frailty scores but not necessarily be different between the educational or income groups. Improvements in diagnostics may account for part of the observed overall increase in frailty over time,^28^ though this is unlikely to affect the observed trends regarding SES. Additionally, the HFRS was developed on the cumulative deficit model of frailty, which inherently overlaps with multimorbidity, and other measures of frailty (eg, frailty phenotype models)^29^ might show slightly different trends over time. Overall, this likely led to an underestimation in frailty, but it is unclear if this underestimation differs by population subgroup. Though we sought to account for this by investigating the HFRS only among those who sought care in inpatient and outpatient settings (ie, non-zero) and these estimates are likely a more valid reflection of frailty. Finally, this study was conducted in a country with universal healthcare, so our findings, particularly those relating to the equalisation of socioeconomic groups over time, may not be directly generalisable to other types of healthcare systems.

While we have shown that frailty in this population is becoming more common while the relationship between frailty and mortality has remained relatively stable,^4^ this current study shows that there was a trend toward equalisation between SES groups from 1990 to 2020. This trend towards more equitable distribution, particularly between men and women and educational levels, may be indicative of the improvement of health disparity. Differences in frailty between groups may indicate the existence of compositional influences, although this needs to be further investigated. Regardless, because frailty is on the rise across all SES strata, additional research into care and how to best support the ageing population is needed. This type of research could provide critical information for health workers and policy-makers looking to reduce frailty for the benefit of frail patients as well as the long-term viability of the healthcare system.

## Data Availability

Data are available upon reasonable request. Data may be obtained from a third party and are not publicly available. Due to the General Data Protection Regulation in Sweden, the pseudoanonymised personal data underlying this study cannot be shared publicly. Access to the data and the codes for data analyses can be permitted to external researchers after ethical vetting and establishment of a collaboration agreement. Contact the corresponding author (AW) for questions about data sharing.
