# Supplementary material for: Population frailty trends by education and income levels over a period of 30 years: findings from Swedish registry data
Source: J Epidemiol Community Health. 2023 Oct 3;78(2):109–14. doi: 10.1136/jech-2023-221060 (PMC10850722; doi:10.1136/jech-2023-221060)
Supplement: Supplementary data [file jech-2023-221060supp001.pdf]

Supplementary Table 1. Swedish ICD 9 and 10 codes used to create the HFRS

ICD9	ICD10	ICD DESCRIPTION	WEIGHT
331A	F00	Dementia in Alzheimer's disease	7.1
342X	G81	Hemiplegia	4.4
331A	G30	Alzheimer's disease	4.0
438	I69	Sequelae of cerebrovascular disease	3.7
781X	R29	Other symptoms and signs involving the nervous and musculoskeletal systems	3.6
596X	N39	Other disorders of urinary system (includes urinary tract infection and urinary incontinence)	3.2
293	F05	Delirium, not induced by alcohol and other psychoactive substances	3.2
E888	W19	Unspecified fall	3.2
910W	S00	Superficial injury of head	3.2
599H	R31	Unspecified haematuria	3.0
041	B96	Other bacterial agents as the cause of diseases classified to other chapters	2.9
799X	R41	Other symptoms and signs involving cognitive functions and awareness	2.7
781C	R26	Abnormalities of gait and mobility	2.6
437X	I67	Other cerebrovascular diseases	2.6
780D	R56	Convulsions, not elsewhere classified	2.6
780A	R40	Somnolence, stupor and coma	2.5
996H	T83	Complications of genitourinary prosthetic devices, implants and grafts	2.4
854B	S06	Intracranial injury	2.4
812A	S42	Fracture of shoulder and upper arm	2.3
276	E87	Other disorders of fluid, electrolyte and acid-base balance	2.3
719	M25	Other joint disorders, not elsewhere classified	2.3
276	E86	Volume depletion	2.3
797	R54	Senility	2.2
V57W	Z51	Care involving use of rehabilitation procedures	2.1
294B	F03	Unspecified dementia	2.1
E885	W18	Other fall on same level	2.1
V63X	Z75	Problems related to medical facilities and other health care	2.0
290E	F01	Vascular dementia	2.0
916	S80	Superficial injury of lower leg	2.0
681	L03	Cellulitis	2.0
369	H54	Blindness and low vision	1.9
266C	E53	Deficiency of other B group vitamins	1.9
V62W	Z60	Problems related to social environment	1.8
332A	G20	Parkinson's disease	1.8
780C	R55	Syncope and collapse	1.8
807	S22	Fracture of rib(s), sternum and thoracic spine	1.8
564X	K59	Other functional intestinal disorders	1.8
584X	N17	Acute renal failure	1.8
707A	L89	Decubitus ulcer	1.7
V02	Z22	Carrier of infectious disease	1.7
041A	B95	Streptococcus and staphylococcus as the cause of diseases classified to other chapters	1.7
707B	L97	Ulcer of lower limb, not elsewhere classified	1.6
799W	R44	Other symptoms and signs involving general sensations and perceptions	1.6
532	K26	Duodenal ulcer	1.6
458X	I95	Hypotension	1.6

586	N19	Unspecified renal failure	1.6
038X	A41.9	Other septicaemia	1.6
V13	Z87	Personal history of other diseases and conditions	1.5
518W	J96	Respiratory failure, not elsewhere classified	1.5
V01X	X59	Exposure to unspecified factor	1.5
715	M19	Other arthrosis	1.5
345	G40	Epilepsy	1.5
733A	M81	Osteoporosis without pathological fracture	1.4
821A	S72	Fracture of femur	1.4
808W	S32	Fracture of lumbar spine and pelvis	1.4
251	E16	Other disorders of pancreatic internal secretion	1.4
794	R94	Abnormal results of function studies	1.4
585	N18	Chronic renal failure	1.4
788C	R33	Retention of urine	1.3
799X	R69	Unknown and unspecified causes of morbidity	1.3
593X	N28	Other disorders of kidney and ureter, not elsewhere classified	1.3
788D	R32	Unspecified urinary incontinence	1.2
331X	G31	Other degenerative diseases of nervous system, not elsewhere classified	1.2
136X	Y95	Nosocomial condition	1.2
959A	S09	Other and unspecified injuries of head	1.2
799C	R45	Symptoms and signs involving emotional state	1.2
435	G45	Transient cerebral ischaemic attacks and related syndromes	1.2
V60X	Z74	Problems related to care-provider dependency	1.1
729X	M79	Other soft tissue disorders, not elsewhere classified	1.1
E884	W06	Fall involving bed	1.1
873A	S01	Open wound of head	1.1
008W	A04	Other bacterial intestinal infections	1.1
009D	A09	Diarrhoea and gastroenteritis of presumed infectious origin	1.1
486	J18	Pneumonia, organism unspecified	1.1
507	J69	Pneumonitis due to solids and liquids	1.0
784F	R47	Speech disturbances, not elsewhere classified	1.0
268X	E55	Vitamin D deficiency	1.0
V44	Z93	Artificial opening status	1.0
785E	R02	Gangrene, not elsewhere classified	1.0
783X	R63	Symptoms and signs concerning food and fluid intake	0.9
389X	H91	Other hearing loss	0.9
E880	W10	Fall on and from stairs and steps	0.9
E885	W01	Fall on same level from slipping, tripping and stumbling	0.9
242X	E05	Thyrotoxicosis [hyperthyroidism]	0.9
737D	M41	Scoliosis	0.9
787C	R13	Dysphagia	0.8
V46W	Z99	Dependence on enabling machines and devices	0.8
V09	U82.0	Agent resistant to penicillin and related antibiotics	0.8
733A	M80	Osteoporosis with pathological fracture	0.8
536X	K92	Other diseases of digestive system	0.8
434X	I63	Cerebral Infarction	0.8
592B	N20	Calculus of kidney and ureter	0.7
291	F10	Mental and behavioural disorders due to use of alcohol	0.7
E879	Y84	Other medical procedures as the cause of abnormal reaction of the patient	0.7

785B	R00	Abnormalities of heart beat	0.7
519W	J22	Unspecified acute lower respiratory infection	0.7
V62	Z73	Problems related to life-management difficulty	0.6
790G	R79	Other abnormal findings of blood chemistry	0.6
V15X	Z91	Personal history of risk-factors, not elsewhere classified	0.5
881	S51	Open wound of forearm	0.5
296	F32	Depressive episode	0.5
724	M48.0	Spinal stenosis (secondary code only)	0.5
275	E83	Disorders of mineral metabolism	0.4
716F	M15	Polyarthrosis	0.4
285X	D64	Other anaemias	0.4
686X	L08	Other local infections of skin and subcutaneous tissue	0.4
787A	R11	Nausea and vomiting	0.3
558	K52	Other noninfective gastroenteritis and colitis	0.3
780G	R50	Fever of unknown origin	0.1

Supplementary Table 2. Proportion of people from original birth cohort surviving and present in the National Patient Register at the year of study, by age

Age 75					Age 85					Age 95				
Birth year	Study year	Original birth cohort, n	Alive at study year, n (%)	Present in Patient Register, n (%)	Birth year	Study year	Original birth cohort, n	Alive at study year, n (%)	Present in Patient Register, n (%)	Birth year	Study year	Original birth cohort, n	Alive at study year, n (%)	Present in Patient Register, n (%)
1915	1990	122997	70658 (57.4)	6747 (9.5)	1905	1990	135409	32593 (24.1)	6776 (20.8)	1895	1990	134599	3273 (2.4)	1093 (33.4)
1916	1991	121679	72372 (59.5)	7453 (10.3)	1906	1991	136620	36131 (26.4)	8252 (22.8)	1896	1991	134308	4166 (3.1)	1517 (36.4)
1917	1992	120855	72995 (60.4)	7977 (10.9)	1907	1992	136793	37203 (27.2)	8641 (23.2)	1897	1992	132999	4164 (3.1)	1498 (36.0)
1918	1993	117955	71603 (60.7)	7879 (11.0)	1908	1993	138874	38369 (27.6)	8308 (21.7)	1898	1993	136523	4353 (3.2)	1264 (29.0)
1919	1994	115193	71713 (62.3)	8038 (11.2)	1909	1994	139505	39956 (28.6)	8766 (21.9)	1899	1994	133882	4463 (3.3)	1204 (27.0)
1920	1995	138753	87141 (62.8)	10041 (11.5)	1910	1995	135625	40035 (29.5)	8898 (22.2)	1900	1995	138139	4923 (3.6)	1233 (25.0)
1921	1996	127723	82508 (64.6)	9255 (11.2)	1911	1996	132977	40095 (30.2)	8759 (21.8)	1901	1996	139370	5172 (3.7)	1299 (25.1)
1922	1997	116946	76690 (65.6)	8149 (10.6)	1912	1997	132868	41298 (31.1)	8639 (20.9)	1902	1997	137364	5424 (3.9)	1369 (25.2)
1923	1998	113435	76047 (67.0)	7524 (9.9)	1913	1998	130200	41512 (31.9)	8363 (20.1)	1903	1998	133896	5629 (4.2)	1369 (24.3)
1924	1999	109055	74407 (68.2)	7425 (10.0)	1914	1999	129458	41622 (32.2)	8379 (20.1)	1904	1999	134952	5906 (4.4)	1574 (26.7)
1925	2000	106292	73137 (68.8)	7295 (10.0)	1915	2000	122997	40620 (33.0)	8185 (20.2)	1905	2000	135409	6128 (4.5)	1596 (26.0)
1926	2001	102007	71093 (69.7)	8888 (12.5)	1916	2001	121679	41106 (33.8)	9186 (22.3)	1906	2001	136620	6522 (4.8)	1834 (28.1)
1927	2002	97994	69175 (70.6)	10849 (15.7)	1917	2002	120855	42136 (34.9)	11115 (26.4)	1907	2002	136793	6842 (5.0)	2116 (30.9)
1928	2003	97868	70383 (71.9)	11757 (16.7)	1918	2003	117955	41530 (35.2)	10899 (26.2)	1908	2003	138874	6963 (5.0)	2217 (31.8)
1929	2004	92861	67788 (73.0)	11596 (17.1)	1919	2004	115193	41906 (36.4)	10443 (24.9)	1909	2004	139505	7313 (5.2)	2212 (30.2)
1930	2005	94220	69331 (73.6)	12592 (18.2)	1920	2005	138753	51461 (37.1)	13296 (25.8)	1910	2005	135625	7379 (5.4)	2303 (31.2)
1931	2006	91074	67772 (74.4)	12369 (18.3)	1921	2006	127723	49380 (38.7)	13483 (27.3)	1911	2006	132977	7641 (5.7)	2552 (33.4)
1932	2007	89779	67694 (75.4)	12697 (18.8)	1922	2007	116946	46041 (39.4)	13620 (29.6)	1912	2007	132868	8026 (6.0)	2812 (35.0)
1933	2008	85020	64932 (76.4)	12549 (19.3)	1923	2008	113435	46573 (41.1)	14058 (30.2)	1913	2008	130200	8118 (6.2)	2992 (36.9)
1934	2009	85092	65981 (77.5)	13320 (20.2)	1924	2009	109055	45725 (41.9)	14221 (31.1)	1914	2009	129458	8139 (6.3)	3055 (37.5)
1935	2010	85906	67585 (78.7)	13906 (20.6)	1925	2010	106292	45354 (42.7)	14237 (31.4)	1915	2010	122997	8002 (6.5)	3054 (38.2)
1936	2011	88938	70594 (79.4)	14791 (21.0)	1926	2011	102007	44676 (43.8)	14379 (32.2)	1916	2011	121679	8414 (6.9)	3304 (39.3)
1937	2012	90373	72471 (80.2)	15719 (21.7)	1927	2012	97994	43660 (44.6)	14470 (33.1)	1917	2012	120855	8777 (7.3)	3496 (39.8)
1938	2013	93946	76039 (80.9)	16682 (21.9)	1928	2013	97868	45017 (46.0)	15035 (33.4)	1918	2013	117955	8650 (7.3)	3629 (42.0)
1939	2014	97380	78928 (81.1)	17271 (21.9)	1929	2014	92861	43419 (46.8)	14641 (33.7)	1919	2014	115193	8959 (7.8)	3745 (41.8)
1940	2015	95778	78694 (82.2)	17675 (22.5)	1930	2015	94220	45150 (47.9)	15494 (34.3)	1920	2015	138753	11332 (8.2)	4832 (42.6)

1941	2016	99727	82417 (82.6)	18460 (22.4)	1931	2016	91074	44228 (48.6)	15007 (33.9)	1921	2016	127723	10865 (8.5)	4508 (41.5)
1942	2017	113961	93096 (81.7)	20960 (22.5)	1932	2017	89779	44385 (49.4)	15236 (34.3)	1922	2017	116946	10254 (8.8)	4368 (42.6)
1943	2018	125392	101301 (80.8)	22916 (22.6)	1933	2018	85020	43112 (50.7)	14727 (34.2)	1923	2018	113435	10491 (9.2)	4526 (43.1)
1944	2019	134991	108298 (80.2)	24232 (22.4)	1934	2019	85092	43758 (51.4)	14880 (34.0)	1924	2019	109055	10667 (9.8)	4544 (42.6)
1945	2020	135373	110524 (81.6)	23698 (21.4)	1935	2020	85906	45289 (52.7)	15043 (33.2)	1925	2020	106292	10683 (10.1)	4406 (41.2)

Supplementary Table 3. Percentage of sociodemographic groups among the frail population

	Age 75										Age 85										Age 95									
				Education tertile			Income quartile							Education tertile			Income quartile							Education tertile			Income quartile			
Year	Born	Men	W	1st	2nd	3rd	1st	2nd	3rd	4th	Born	Men	W	1st	2nd	3rd	1st	2nd	3rd	4th	Born	Men	W	1st	2nd	3rd	1st	2nd	3rd	4th
1990	1915	43.9	56.1	74.3	17.8	4.5	23.6	24.2	24.2	24.1	1905	34.8	65.2	0.0	0.0	0.0	22.2	21.9	22.8	22.4	1895	23.8	76.2	0.0	0.0	0.0	18.4	18.2	18.8	18.5
1991	1916	44.5	55.5	71.0	20.1	5.8	24.4	24.4	24.7	24.6	1906	35.8	64.2	0.0	0.0	0.0	23.1	23.8	24.0	23.7	1896	27.2	72.8	0.0	0.0	0.0	20.6	21.8	21.6	21.5
1992	1917	44.4	55.6	68.9	21.7	6.6	24.3	24.6	24.7	24.6	1907	35.9	64.1	0.0	0.0	0.0	23.1	24.0	23.6	23.9	1897	25.2	74.8	0.0	0.0	0.0	21.3	21.6	22.0	22.0
1993	1918	44.4	55.6	66.5	23.2	7.4	24.4	24.5	24.7	24.6	1908	35.6	64.4	0.0	0.0	0.0	23.3	23.8	23.7	23.8	1898	24.6	75.4	0.0	0.0	0.0	21.2	21.7	21.5	21.7
1994	1919	44.7	55.3	65.9	23.9	7.6	24.4	24.5	24.7	24.6	1909	36.4	63.6	0.0	0.0	0.0	23.0	23.8	23.8	23.8	1899	24.8	75.2	0.0	0.0	0.0	20.7	21.2	21.3	21.2
1995	1920	44.6	55.4	65.4	24.6	7.2	24.3	24.7	24.6	24.7	1910	36.8	63.2	0.0	0.0	0.0	23.5	23.3	24.0	23.8	1900	23.6	76.4	0.0	0.0	0.0	21.6	21.6	22.0	21.9
1996	1921	44.6	55.4	64.1	25.5	7.6	24.3	24.5	24.7	24.6	1911	36.8	63.2	74.7	15.3	4.6	23.5	23.6	24.1	23.8	1901	24.4	75.6	0.0	0.0	0.0	21.5	21.4	21.6	21.6
1997	1922	44.5	55.5	63.3	25.9	7.9	24.3	24.8	24.4	24.7	1912	36.2	63.8	75.5	15.7	4.5	23.7	23.6	23.8	23.9	1902	23.9	76.1	0.0	0.0	0.0	21.2	22.1	22.1	21.8
1998	1923	45.1	54.9	61.7	27.0	8.4	24.6	24.6	24.6	24.6	1913	36.9	63.1	74.7	16.9	4.8	23.7	23.7	23.8	23.8	1903	23.8	76.2	0.0	0.0	0.0	21.9	21.7	22.0	22.0
1999	1924	44.7	55.3	60.3	27.6	9.1	24.5	24.6	24.6	24.6	1914	36.7	63.3	74.2	17.4	4.9	23.8	23.8	23.8	23.8	1904	23.6	76.4	0.0	0.0	0.0	21.4	21.5	21.7	21.7
2000	1925	44.9	55.1	58.5	28.5	10.0	24.6	24.6	24.6	24.6	1915	36.6	63.4	73.4	18.4	5.1	23.7	23.9	23.8	23.8	1905	23.4	76.6	0.0	0.0	0.0	21.5	21.6	21.5	21.6
2001	1926	44.9	55.1	59.4	28.5	10.8	24.6	24.6	24.7	24.6	1916	37.3	62.7	70.0	20.9	6.5	23.7	23.9	23.8	23.8	1906	23.9	76.1	0.0	0.0	0.0	21.5	21.7	21.8	21.7
2002	1927	44.9	55.1	58.1	29.0	11.6	24.6	24.6	24.6	24.6	1917	37.2	62.8	67.9	22.4	7.3	23.7	23.8	23.8	23.8	1907	24.0	76.0	0.0	0.0	0.0	21.4	21.4	21.5	21.5
2003	1928	44.9	55.1	56.7	29.5	12.5	24.5	24.7	24.6	24.6	1918	37.6	62.4	65.4	24.1	8.1	23.8	23.9	23.8	23.8	1908	23.0	77.0	0.0	0.0	0.0	21.2	21.3	21.2	21.3
2004	1929	45.2	54.8	55.4	30.2	13.0	24.5	24.6	24.6	24.6	1919	38.0	62.0	64.6	24.7	8.3	23.9	23.9	23.9	23.9	1909	24.7	75.3	0.0	0.0	0.0	21.7	21.5	21.8	21.7
2005	1930	45.1	54.9	53.6	30.8	14.0	24.6	24.6	24.6	24.7	1920	38.1	61.9	64.3	25.5	8.0	23.8	23.8	23.9	23.9	1910	24.7	75.3	0.0	0.0	0.0	21.6	21.8	21.8	21.7
2006	1931	45.6	54.4	51.9	31.9	14.9	24.6	24.6	24.6	24.7	1921	38.1	61.9	62.9	26.2	8.5	23.9	23.9	24.0	24.0	1911	24.7	75.2	71.8	16.8	6.0	21.6	21.8	21.7	21.7
2007	1932	45.8	54.2	50.3	32.6	15.7	24.6	24.6	24.7	24.7	1922	38.4	61.6	61.9	26.8	8.8	23.8	23.9	23.8	23.8	1912	24.5	75.5	72.7	17.3	5.8	21.7	21.9	21.9	21.9
2008	1933	46.2	53.8	48.5	33.5	16.5	24.7	24.7	24.7	24.7	1923	39.4	60.6	60.4	27.9	9.2	23.9	24.0	23.9	24.0	1913	25.4	74.6	72.1	18.4	6.1	21.4	21.5	21.6	21.5
2009	1934	46.2	53.8	47.1	34.6	16.8	24.6	24.7	24.7	24.7	1924	38.8	61.2	59.0	28.4	10.1	23.8	24.0	23.9	23.9	1914	26.5	73.5	72.3	18.8	5.6	21.7	21.7	21.8	21.8
2010	1935	47.2	52.8	45.2	35.4	18.1	24.6	24.7	24.7	24.7	1925	39.2	60.8	57.0	29.4	11.0	23.9	24.0	24.0	24.0	1915	25.1	74.9	70.2	20.1	6.6	21.6	21.6	21.7	21.7
2011	1936	46.9	53.1	43.4	35.9	19.3	24.7	24.6	24.7	24.7	1926	39.3	60.7	57.5	29.7	11.8	24.0	24.0	24.0	24.0	1916	26.3	73.7	66.8	22.7	8.0	21.5	21.7	21.6	21.6
2012	1937	47.4	52.6	41.9	36.7	20.1	24.7	24.7	24.7	24.7	1927	39.4	60.6	56.5	29.7	12.7	24.0	24.0	24.0	24.0	1917	26.8	73.2	64.9	24.0	8.6	21.7	21.9	21.8	21.8
2013	1938	47.5	52.5	40.0	37.7	20.9	24.7	24.7	24.7	24.7	1928	40.0	60.0	54.9	30.3	13.7	24.0	24.0	24.0	24.0	1918	27.5	72.5	62.5	25.3	9.8	21.5	21.6	21.6	21.6
2014	1939	48.0	52.0	38.5	38.6	21.6	24.7	24.7	24.7	24.7	1929	40.2	59.8	53.4	31.0	14.5	23.9	24.1	24.0	24.1	1919	27.5	72.5	61.1	26.8	9.8	22.1	22.0	22.1	22.1

2015	1940	48.4	51.6	36.7	39.7	22.1	24.7	24.7	24.7	24.7	1930	40.5	59.5	51.6	31.6	15.4	23.9	24.1	24.0	24.0	1920	27.7	72.3	61.0	26.9	9.8	21.7	21.7	21.8	21.8
2016	1941	48.4	51.6	34.5	40.6	23.7	24.7	24.7	24.7	24.7	1931	40.9	59.1	50.0	32.5	16.4	24.1	24.1	24.1	24.1	1921	28.2	71.8	59.6	27.6	10.3	22.0	21.8	22.2	22.0
2017	1942	48.8	51.2	31.9	41.0	26.0	24.7	24.7	24.7	24.7	1932	41.6	58.4	48.3	33.1	17.4	24.0	24.1	24.0	24.1	1922	28.7	71.3	58.3	28.7	10.5	21.6	21.5	21.8	21.6
2018	1943	48.6	51.4	30.1	42.0	26.8	24.7	24.7	24.7	24.7	1933	41.9	58.1	46.4	34.2	18.1	23.9	24.1	24.0	24.1	1923	29.4	70.6	56.9	29.4	11.2	21.8	21.9	21.9	21.9
2019	1944	48.9	51.1	29.0	42.5	27.5	24.7	24.7	24.7	24.7	1934	41.9	58.1	44.8	35.2	18.5	24.1	24.1	24.1	24.1	1924	29.0	71.0	56.2	30.1	11.7	21.9	22.0	22.0	22.0
2020	1945	48.9	51.1	28.5	42.5	27.9	24.7	24.7	24.8	24.7	1935	43.2	56.8	43.0	36.0	19.8	24.0	24.2	24.1	24.1	1925	29.2	70.8	53.3	30.8	13.6	22.0	22.1	22.1	22.1

BMJ Publishing Group Limited (BMJ) disclaims all liability and responsibility arising from any reliance
placed on this supplemental material which has been supplied by the author(s)

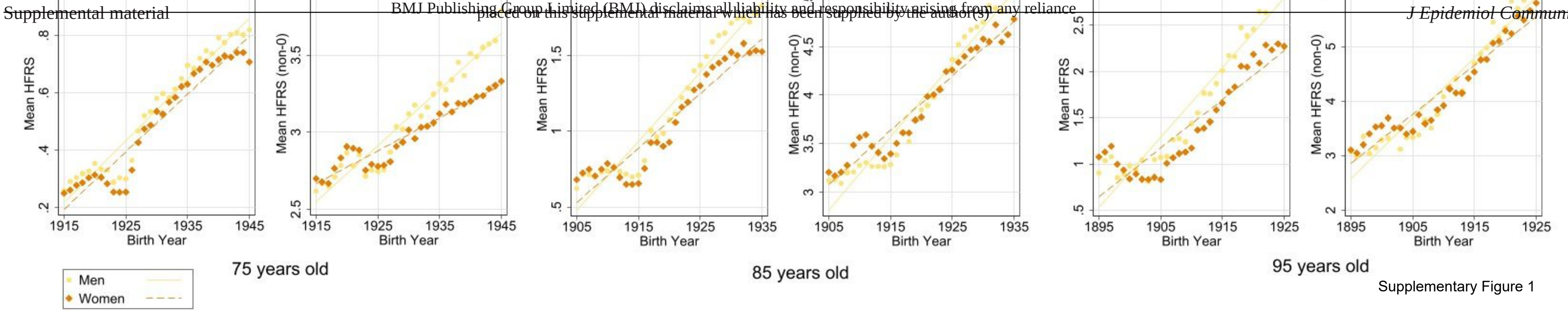

Supplementary Figure 1

BMJ Publishing Group Limited (BMJ) disclaims all liability and responsibility arising from any reliance
placed on this supplemental material which has been supplied by the author(s)

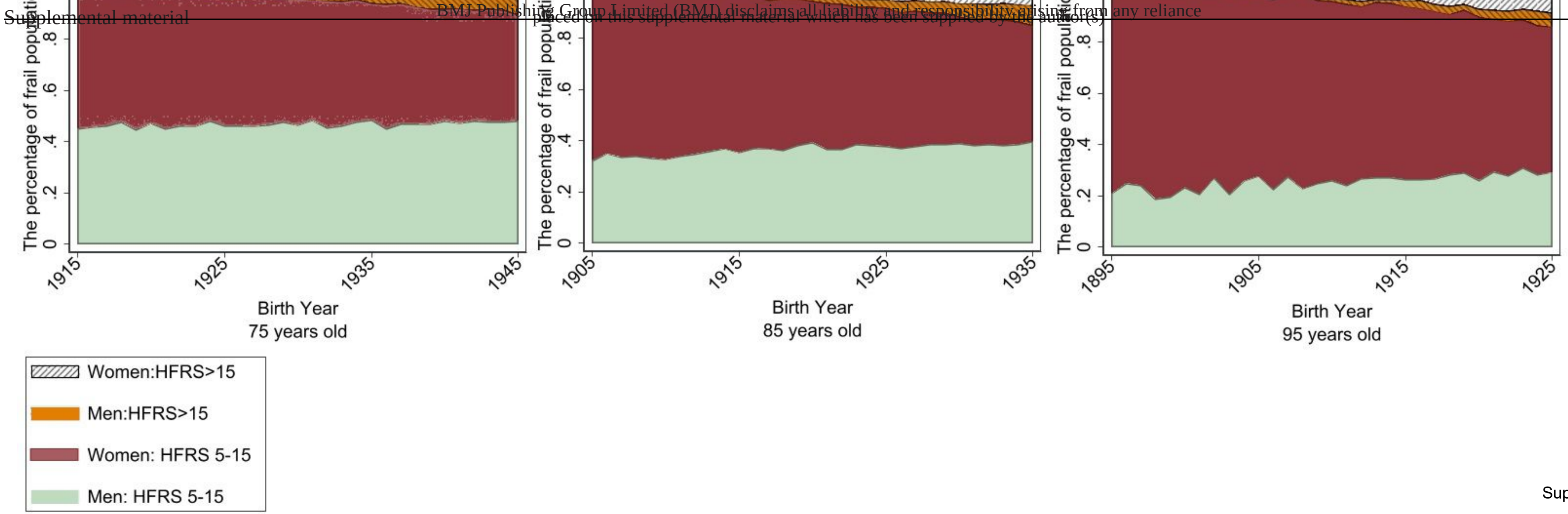

Supplementary Figure 2

BMJ Publishing Group Limited (BMJ) disclaims all liability and responsibility arising from any reliance placed on this supplemental material which has been supplied by the author(s)

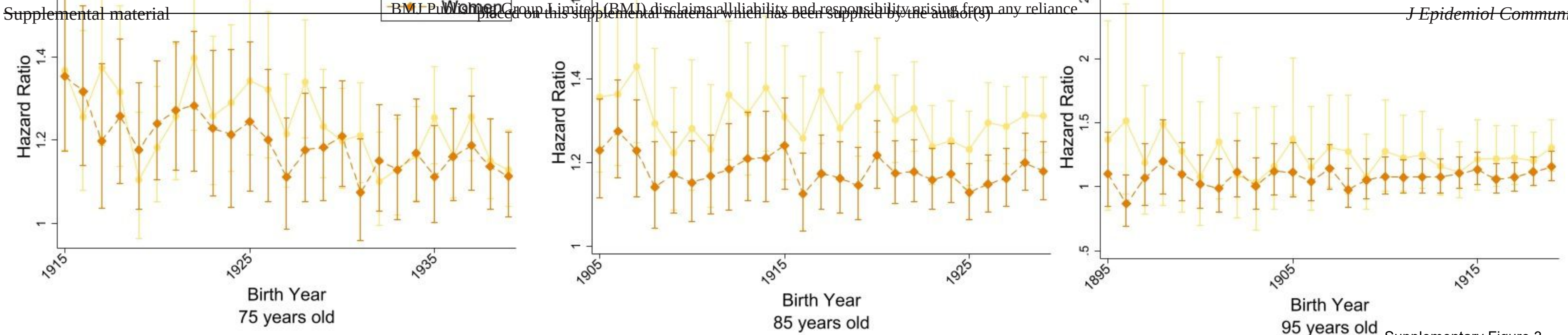

Supplementary Figure 3

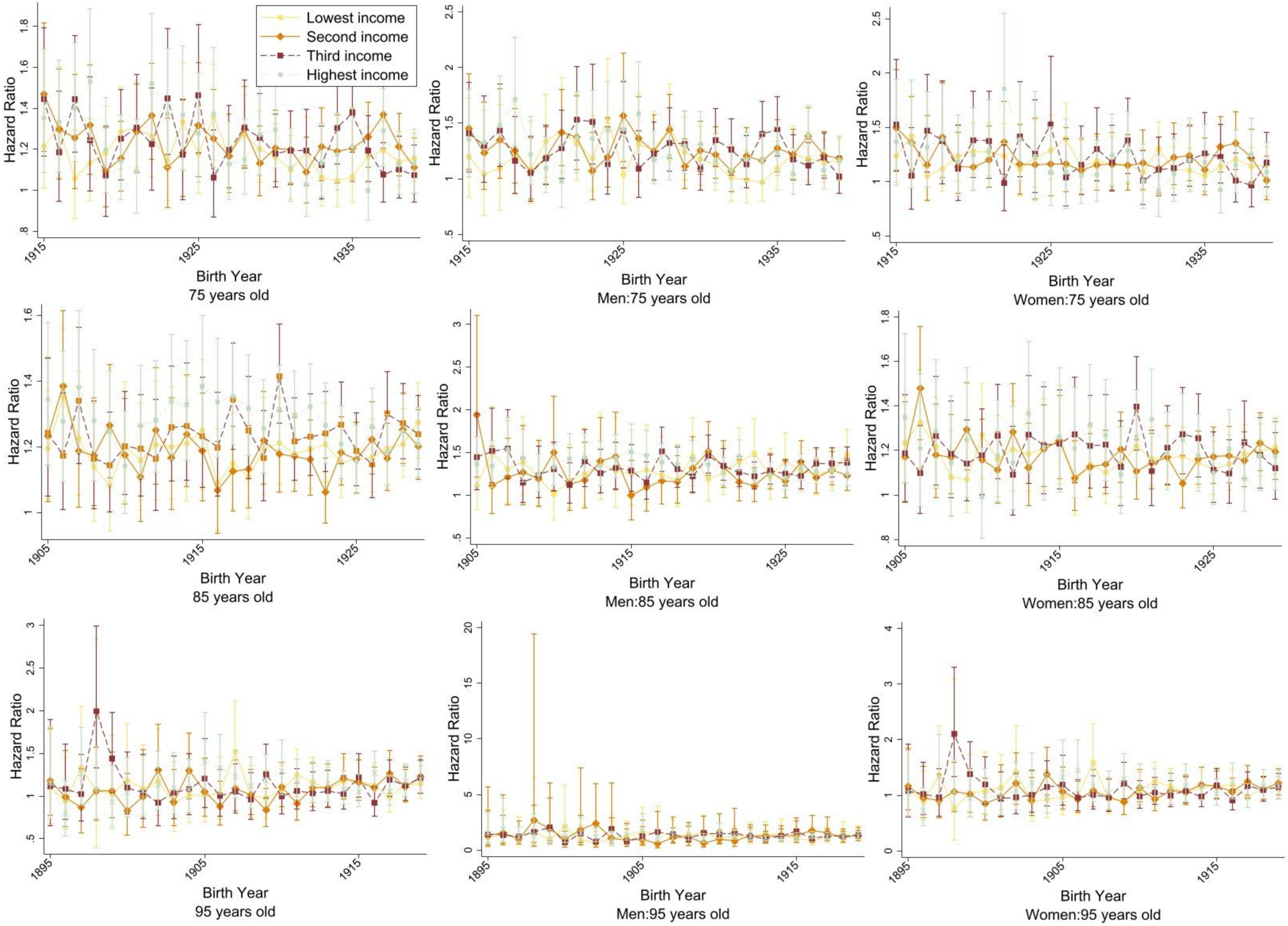

Supplementary Figure 4
